# The Quality of Counseling for Headache OTC Medications in German Community Pharmacies Using a Simulated Patient Approach: Are There Differences between Self-Purchase and Purchase for a Third Party?

**DOI:** 10.1155/2022/5851117

**Published:** 2022-03-23

**Authors:** Bernhard Langer, Christian Kunow

**Affiliations:** Department of Health, Nursing, Management, University of Applied Sciences Neubrandenburg, Neubrandenburg, Germany

## Abstract

**Background:**

In Germany—as worldwide—headache is one of the most frequent causes of self-medication. The dispensing of over-the-counter (OTC) medications may only be carried out by community pharmacies (CPs). In doing so, CPs have to ensure “adequate” counseling, for both self-purchase and purchase for a third party, which also occurs in everyday pharmacy practice. The aim of this study was to evaluate the quality of counseling for headache OTC medications in German CPs and, as the first study worldwide, to analyze whether and to what extent there are differences in counseling between self-purchase and purchase for a third party.

**Methods:**

A cross-sectional study was carried out using the covert simulated patient methodology (SPM) in all 42 CPs in the German big city Potsdam. With the help of 8 trained simulated patients (SPs), each CP was visited four times by a different SP. The SPs simulated in each CP two scenarios two times with the demand for an OTC medication against headache, which differed only in whether the demand was for themselves or for their boyfriend/girlfriend.

**Results:**

All 168 planned pharmacy visits (84 visits per scenario) were successfully carried out. Overall, the median counseling score was 3.0 out of 9 points (interquartile range [IQR 2.0]). There were no significant differences between the two scenarios (Wilcoxon signed-rank test; *p*=0.495, *r* = 0.053). In a multivariate binary logistic regression analysis, the counseling level and the different scenarios were not significantly associated (adjusted odds ratio [AOR] = 1.635, 95% CI = 0.673–3.972, *p*=0.278).

**Conclusions:**

Due to the partly considerable deficits in counseling of German CPs, policy-makers and the regional chambers of pharmacists are called upon to take appropriate measures to improve the quality of counseling. It is positive that no differences in counseling between self-purchase and purchase for a third party were found, but further verifying studies with a modified methodology are recommended.

## 1. Introduction

Headache disorders, especially migraine and tension-type headache (hereafter referred to as headache), are not only among the most prevalent disorders worldwide, but they are also the leading causes of years of life affected by disease [1]. In Germany, for example, there are over 35 million cases of headache each year and over 700,000 years of life spent with it [2]. Thus, since headache is a public health challenge in addition to its associated costs [3, 4] and also belongs to minor ailments [5, 6], its medication treatment is basically possible without a medical consultation. In this regard, headache is one of the most common causes of self-medication worldwide [7, 8] and thus also in Germany [9]. For medication treatment, the guidelines for Germany [10, 11] recommend appropriate analgesics, which—analogous to other countries [12, 13]—are also available without prescription.

In contrast to some other countries [14], such over-the-counter (OTC) medications may only be dispensed by community pharmacies (CPs) in Germany [15]. Against the background of the worldwide role of CPs as “epidemiological sentinel” [16] of headache, the German CPs have to ensure an “adequate” counseling [17]. This must be provided by pharmacists, but can also be provided by nonpharmacists (pharmacy technicians and pharmaceutical technical assistants) if the pharmacy manager has previously specified this [17]. The Federal Chamber of Pharmacists (BAK) has drafted a tool for self-medication for headache, among other things, for the quality assurance of counseling [18]. On the one hand, compliance with the tool by the pharmacy staff is intended to ensure that the customer is first asked appropriate questions to obtain information, the answers to which are decisive for a possible recommendation of a suitable OTC analgesic. On the other hand, it should be ensured that the customer is then provided with the appropriate information if the product is dispensed. In addition to the legal obligation, the need for “adequate” counseling could also arise from existing knowledge deficits or incorrect knowledge about OTC analgesics among the population in Germany [19]. With regard to an assessment of the counseling quality of German CPs on self-medication against headache, the studies known to the authors [20, 21] are outdated. Therefore, an up-to-date study—as it is already available for other indications [22–24]—is indicated.

Regarding possible differences in the quality of counseling, numerous possible influencing factors have already been investigated worldwide for various indications, such as person of the customer [25], gender of the customer [26], age of the customer [27], type of request (symptom-based vs. medication-based) [28], time of request [29], queue at the pharmacy visit [30, 31], professional group of the pharmacy staff [32], age of the pharmacy staff [32], gender of the pharmacy staff [33], number of questions asked of the pharmacy staff [34], pharmacy location [25, 35], socioeconomic status of the pharmacy location [36, 37], pharmacy type [35, 37], pharmacy size [36], CP quality certificate [37], private counseling area [26], types of medication [29], and price of dispensed medicines [38].

With regard to the question of who the purchase is for, the only information known to date is that purchases both for oneself (self-purchase) and for a third party (purchase for a third party) occur in everyday pharmacy practice [39–41]. A recent customer survey specifically on OTC supply by Australian CPs concluded that 24.5% of respondents were third-party customers [40]. In an older study from Uganda, this proportion was as high as 32.3% [41]. According to national [42] and international guidelines [43], the quality of counseling for a purchase for a third party should be identical to a self-purchase. The reason for this is that a high quality of counseling should also be ensured for the patient who is not present in the CP. On the one hand, this includes appropriate information gathering by the pharmacy staff, but this is only possible if the patient passes on information about his or her health status (e.g., how often the symptoms occur) to the third-party customer, who then requests the drug from the pharmacy on behalf of the patient, and the third-party customer also communicates the corresponding information to the pharmacy staff. Conversely, it is necessary that the information given by the pharmacy staff (e.g., about duration of the dispensed drug) is also communicated to the patient by the third-party customer. Thus, from a theoretical point of view, the communication process for the purchase for a third party is much more complex than for the self-purchase, which makes it more difficult to ensure adequate quality of counseling. This problem is also suspected by German [30, 44] and international authors [41, 45] in everyday pharmacy practice. However, to the authors' knowledge, explicit studies investigating the current everyday pharmacy practice are not yet available worldwide.

The aim was to evaluate the quality of counseling for headache OTC medications in German CPs and to analyze whether and to what extent differences exist between self-purchase and purchase for a third party.

## 2. Materials and Methods

### 2.1. Design

The cross-sectional study was based on the internationally widely used [46, 47] simulated patient methodology (SPM) as a form of covert participatory observation [48]. Here, a person, who in an ideal case, is indistinguishable from a real customer, visits a CP to simulate a real-life counseling situation based on a previously defined scenario. The data are then collected on the basis of previously defined criteria using an assessment form, and the CP is provided with performance feedback, if applicable [46]. Although terms such as “pseudo customer,” “simulated client,” or “mystery shopper” apply to both self-purchase and purchase for a third party, the term “simulated patient” (SP) is used for the putative customer in the following, as it is the most common internationally [47]. The study is reported according to the “STROBE Statement–Checklist of items that should be included in reports of cross-sectional studies” [49] and, based on this, according to the “Checklist for Reporting Research Using Simulated Patient Methodology” (CRiSP) [50].

### 2.2. Setting and Participation

All 42 CPs of the state capital Potsdam (31.12.2020: 182,112 inhabitants; ranked #42 among the biggest German cities) [51] of the German federal state Brandenburg were included, which could be determined on the reference date of 01 September 2020 using the online pharmacy finder of the regional chamber of pharmacists for Brandenburg [52]. These hits were subsequently validated on the one hand using the nationwide pharmacy finder of the Internet portal “Apotheken-Umschau” [53] and on the other hand using a Google search and then confirmed. The visits took place between 19 October and 05 December 2020. To carry out the visits, a total of €673.01 was required, which was financed from the primary author's own resources.

### 2.3. Scenario and Assessment

The BAK tool for self-medication for headache [18] formed the basis for the two scenarios (see Tables 1 and 2) [27] and for the assessment form (see Table 3). The two scenarios are designed as “normal” scenarios so that the limits of self-medication, that is, towards a possible recommendation of a physician visit, should not be exceeded by the pharmacy staff. For example, when asked by the pharmacy staff, the SPs had to state symptoms of only mild and not chronic tension-type headache according to the International Classification of Headache Disorders (ICHD) [54] without other symptoms, without other medical conditions, and without the previous use of other medications. Therefore, counseling provided for this purpose and the recommendation and dispensing of an appropriate OTC analgesic should always occur. This created the prerequisite to be able to check as comprehensively as possible whether the pharmacy staff not only asks questions, for example, about the headache indicated by the SPs, but also provides information about the OTC analgesic that may have been recommended and dispensed.

The only difference between the two scenarios was whether the demand for an OTC analgesic was for the SP oneself (self-purchase, see Table 1) or for the SP's boyfriend/girlfriend (purchase for a third party, see Table 2). Otherwise, the scenarios were identical with regard to the information to be provided by the SP in response to questions from the pharmacy staff. To ensure that the pharmacy staff could know in all visits for whom the requested OTC analgesic was intended and to be able to distinguish between self-purchase and purchase for a third party for all visits, the SPs had to provide the relevant information at the beginning of the consultation. In principle, the SPs should describe their concerns to the pharmacy staff who approached them first and should only provide further information if asked, in order to ensure that the information given is consistent.

The assessment form comprised a total of 9 objective items, the fulfillment of which was determined exclusively on the basis of dichotomous scales. The first 6 items assessed whether appropriate questions were asked by the pharmacy staff. On the basis of these questions, the pharmacy staff had to decide whether an OTC analgesic should be recommended and finally dispensed. In the case of dispensing, it was also assessed whether the respective SPs were given information about dosage, duration, and side effects (7th to 9th items).

### 2.4. Data Collection

A total of 8 people from the Department of Health, Nursing, Management of the University of Applied Sciences Neubrandenburg, aged between 23 and 48 years, acted as SPs: 5 master's students (including 4 women and 1 man), 1 bachelor's student, 1 former master's student, and the project leader, who was highly experienced in SPM. The master's students were selected based on their participation in a 3-semester research project and the bachelor's student was selected based on her completion of her internship semester. The project leader has had intensive research contacts with the former master's student for years.

Before starting the data collection, the 7 student SPs familiarized themselves with the theoretical basics of SPM as well as with the initially planned medication-based scenarios using the OTC analgesic paracetamol and with the assessment form. Subsequently, each of the student SPs performed 4 validation visits with 2 visits for self-purchase and 2 visits for purchase for a third party (4 validation visits × 7 SPs = 28 validation visits) to check the functionality of the planned scenarios and the assessment form and to ensure that the SPs could practice using the SPM. After the validation visits, a workshop was held to share experiences and to inform each other about the specifics of the scenarios and the assessment form. However, it became apparent that the medication-based scenarios were so poorly advised that differences between self-purchase and purchase for a third party could not have been determined. Due to this, symptom-based scenarios were now planned, which, according to findings in the international literature [28, 54], can be expected to result in a higher level of counseling overall compared to medication-based scenarios, so that any differences between self-purchase and purchase for a third party also become more “visible.” Afterwards, each of the student SPs again performed 2 validation visits with 1 visit for self-purchase and 1 visit for purchase for a third party (2 validation visits × 7 SPs = 14 validation visits) with the result that the functionality of the symptom-based scenarios could now be confirmed. A total of 42 validation visits were conducted in different CPs outside Potsdam.

The distribution of the CPs subsequently attended in Potsdam to the respective SPs was carried out by means of the random principle. The random procedure was designed to ensure that no CP was attended more than once by an SP. After the student SPs were no longer allowed to enter Potsdam in December 2020 due to the Corona pandemic, only the former master's student and the project leader conducted the remaining 42 visits (of the third-party purchase scenario), contrary to the original project planning. As before, it was ensured that no CP was visited more than once by an SP.

Each CP was visited 2 times with the self-purchase scenario and the purchase for a third-party scenario, that is, a total of 4 times (4 visits per CP × 42 CPs = 168 visits = 84 visits per scenario). In relation to all CPs visited, there were thus a total of 4 runs (42 visits × 4 runs = 168 visits), whereby each run was always carried out in a period of one week. The 4 runs alternated with regard to the two scenarios; that is, run 1 with the self-purchase scenario was followed by run 2 with the purchase for a third-party scenario, then run 3 with the self-purchase scenario, and finally run 4 with the purchase for a third-party scenario. There was a one-week break between each run to avoid simulating 2 almost identical scenarios in the same CPs too soon after each other and thus increasing the risk of detection. The visits were conducted on different days of the week and at different times of the day. In order to avoid medication waste and corresponding costs, a purchase termination was to take place during the visits, but this was discarded as the majority of SPs stated during the evaluation of the validation visits that they had felt uncomfortable with the respective purchase termination simulation.

In addition to the items of the assessment form, the SPs collected the respective scenario type (self-purchase vs. purchase for a third party) and, analogous to the international literature, also numerous control variables before, during, and after the visits (see Table 4), which may also have an influence on the quality of counseling in addition to the scenario type.

After the evaluation of the data, each CP received written, pharmacy-specific performance feedback, including graphically prepared benchmarking, while preserving the anonymity of the other CPs presented. This provided each CP with information about its competitive position, so that ideally—if necessary—appropriate optimization processes can be initiated by the CPs studied with the aim of sustainably improving the quality of counseling.

### 2.5. Data Management and Analysis

Data were entered using the four-eye principle and analyzed with SPSS version 26 for Windows (IBM, Armonk, NY, USA). The dichotomous 6 question items and 3 information items were summed into a counseling score (min: 0 points; max: 9 points). In addition, the counseling score was dichotomized into a counseling level with the characteristic values “below average” and “above average” by using the demarcation threshold formula (((total highest score–total lowest score)/2) + total lowest score) [56, 57]. Descriptive statistics determined frequencies and percentages for categorical data. Both the Kolmogorov–Smirnov test and the Shapiro–Wilk test showed that the continuous data were not normally distributed. Therefore, the median, interquartile range [IQR], and min. and max. were presented, and in addition, the mean and standard deviation (SD) were also reported in the results tables for better illustration.

Moreover, because of the repeated measurements (4 visits in the same CPs), these were connected samples. Therefore, for categorical variables, the McNemar test and the McNemar–Bowker test were applied to determine correlations. If the McNemar–Bowker test results were significant, post-hoc tests and a Bonferroni–Holm adjustment were performed. The effect sizes of the McNemar test and the McNemar–Bowker test were measured in each case on the basis of Cohen's *g*, whereby according to Cohen, there are a small effect from 0.05, a medium effect from 0.15, and a large effect from 0.25 [58]. For continuous data, the nonparametric Wilcoxon signed-rank test was used to analyze whether differences in the counseling score exist between the two scenarios (self-purchase vs. purchase for a third party). The effect size was measured by the Pearson correlation coefficient *r*, whereby according to Cohen, from 0.10, a small effect, from 0.30, a medium effect, and from 0.50, a large effect are present [58].

A binomial logistic regression analysis was used to determine the association between the counseling level and the different scenarios (self-purchase vs. purchase for a third party) with adjustment for control variables (CP quality certificate, gender and age of the SPs, gender, age, and professional group of the pharmacy staff, time of the visit, queue, and active ingredient) [59, 60]. All independent variables were checked for outliers and multicollinearity. Possible confounding variables with a *p* value less than 0.05 in the univariate analysis were included in the multivariate analysis. Odds ratios (OR), 95% confidence intervals, and *p* values were reported. The measurement of the effect size was based on Cohen's *f*^2^, whereby according to Cohen, from 0.02, a small effect, from 0.15, a medium effect, and from 0.35, a large effect are present [58]. A *p* value of less than 0.05 was considered to be significant in all analyses.

### 2.6. Ethical Approval

The study protocol was approved by the institutional ethics committee of the University of Applied Sciences Neubrandenburg (Registration number: HSNB/166/20). According to the “Guideline for the use of mystery research in market and social research” [61], the data collected were anonymized and recorded in such a way that the CPs or the personnel involved could not be identified. CPs were not asked for consent prior to the study being conducted—analogous to the international literature [46]—because obtaining written consent would have significantly and negatively impacted the results (possible Hawthorne effect [62] and also a possible selection bias [63]). To resolve the issue of informed consent, analogous to recommendations in the international literature [63] and to implementation in numerous studies (e.g., [64–66]), a letter was sent to all selected CPs in advance of study conduct, providing information about the background and conduct of the study. However, specific information about the scenarios used was not provided so as not to compromise the covert study design. For the same reason, an appropriately long period of time (“visits will take place in 2020”) was given in this letter instead of a specific date for conducting the visits. Recruited persons provided their written informed consent to act as SPs.

## 3. Results

Table 5 shows the characteristics of CPs, SPs, pharmacy staff, and visits subdivided by scenario type. In the purchase for a third-party scenario, there was significantly a queue more frequently (McNemar test; *p*=0.029, *g* = 0.194, “medium” effect size according to Cohen [58]), the use of male SPs (McNemar test; *p* < 0.001, *g* = 0.375, “large” effect size according to Cohen [58]), and the use of older SPs (McNemar test; *p* < 0.001, *g*= 0.389, “large” effect size according to Cohen [58]). In addition, there was a significant relationship between scenario type and time of visit (McNemar—Bowker test; *p*=0.003). Post-hoc analyses showed that in the evening (4:01 p.m.–8:00 p.m.) compared to the afternoon (12:01 p.m.–4:00 p.m.), the purchase for a third-party scenario was applied significantly more often (McNemar test; *p*=0.001, *g* = 0.318, “large” effect size according to Cohen [58]).

Medication was dispensed at all visits, with exactly one medication dispensed per visit (168 medications in total). The dispensing behavior (active ingredient, package size, original vs. generic drug, single drug substance vs. fixed-dose combination, recommended vs. not recommended drug, price) and its economic impact were published elsewhere [27].

Overall, the median counseling score was 3.0 [IQR 2.0] with a minimum score of 0 in 6.0% (10/168) of visits and a maximum score of 8 in 0.6% (1/168) of visits. There were no significant differences between the two scenarios. The most frequently asked question was “Are there other medical conditions?” (53.0%, 89/168). The least frequent question was “How long have the symptoms been present?” (10.1%, 17/168). In 73.8% (124/168) of all visits, information was provided regarding the dosage of the medicine. In contrast, the pharmacy staff provided information regarding possible side effects in only 5.4% (9/168) of all visits. There were no significant differences between the two scenarios for the individual question items. For the individual information items, only the item “Information about duration given” showed that in the purchase for a third-party scenario, such information was given significantly less often (McNemar test; *p*=0.003, *g* = 0.244, “medium” effect size according to Cohen [58]) (see Table 6).

Table 7 shows the binomial logistic regression model. “Below-average” counseling occurred in 79.8% (134/168) of all visits, whereas “above-average” counseling occurred in 20.2% (34/168) of visits. Bivariate analysis revealed no significant associations between counseling level and the different scenarios (self-purchase vs. purchase for a third party) (COR = 0.863, 95% CI = 0.406–1.883, *p*=0.701). Three (gender of the SP, age of the SP, and CP quality certificate) of nine control variables had a *p* value < 0.05 in the bivariate analysis and were included in the multivariate logistic regression model. After adjustment for these control variables, there was still no significant association between the counseling level and the different scenarios (self-purchase vs. purchase for a third party) (AOR = 1.635, 95% CI = 0.673–3.972, *p*=0.278). The model yielded a Nagelkerke *R*^2^ value of 0.117, corresponding to Cohen's *f*^2^ of 0.133 and thus a “small” effect size [58].

## 4. Discussion

### 4.1. Counseling Deficits, Their Reasons, and Interventions for Improvement

The rather low level of counseling determined here basically confirms the previous results of other national SPM studies. For example, a fairly recent SPM study for acute diarrhea OTC medications and the associated follow-up study determined a strikingly similar low mean overall score of 3.3 out of 9 points [55] and 2.7 out of 9 points [67], respectively. Counseling deficits were also noted by the 2 most recent national SPM studies: one also for acute diarrhea OTC medications [22] and the other for oral emergency contraception (EC) OTC medications [23]. In addition to contrasting national results from surveys of pharmacy staff [68, 69] and from a passers-by survey [70]—although these results are not surprising due to a social desirability bias or a self-report bias—a nonparticipant observation of pharmacy staff of German CPs for cross-indication counseling practices also found deficits [69]. In any case, with few exceptions [71], the present results are consistent with those of international SPM studies for headache medications [72–75] and for specific analgesics [28, 35, 76].

With regard to the individual assessment items, counseling was quite heterogeneous in the present SPM study. For example, the pharmacy staff asked the question about other medical conditions most frequently, which is initially surprising, as most national studies on other indications [22, 23, 55, 67] or across indications [69] found significantly worse results for this item. In contrast, the question of the duration of symptoms was the least frequently asked, with most national studies identifying less poor outcomes for this item [22, 55, 67], with one exception [69]. These contrasting national results—besides the different indications and scenarios [34] or methods (SPM vs. nonparticipant observation)—could also be explained by the different federal states as study sites. For example, each state has its own chamber of pharmacists, and each chamber initiates different and differently designed measures to ensure the quality of counseling. In any case, most of the international SPM studies specifically on headache medications or on concrete analgesics have found similar results with regard to the question items [28, 35, 71–74] as the present SPM study.

With respect to the information items, dosage was the most frequently advised item by a wide margin. This is confirmed by the results of most national [22, 55, 67] but not international [35, 72–75] SPM studies. However, the national results are not surprising, as information on dosage has the highest priority in counseling for the pharmacy staff of German CPs [68, 69]. On the other hand, the least information was provided about side effects, which was confirmed by almost all studies used for comparison [22, 28, 35, 55, 67, 69, 71–75]. The reason for this uniform study situation could be due to the fact that for the pharmacy staff corresponding information—as documented for the pharmacy staff of German CPs [68, 69]—plays a rather minor role. This can be attributed to the fact that the pharmacy staff may fear a lack of patient adherence as well as loss of sales [69].

In the international literature, lack of time, manpower, interest, and knowledge of the pharmacy staff are seen as possible reasons for the generally rather poor quality of counseling [26, 28]. However, no such evidence is known for the pharmacy staff of German CPs. In an older study, however, pharmacy staff expressed concern that customers might feel patronized when advice is given [20]. In more recent studies, the majority of pharmacy staff stated that most customers—sometimes due to time constraints or preexisting knowledge about the medication—do not want counseling or have already been informed by the physician or in the hospital [68, 69]. However, a recent passers-by survey found that most respondents wanted counseling, even if they did not ask for it directly [70].

For a possible improvement of the quality of counseling, various interventions such as training [77], supportive supervisions [78], repeated sequential verbal feedback loops [34], distribution of educational pamphlets to CPs [79], and implementation and monitoring of guidelines [80] are discussed internationally. In this context, counseling should be trained in Germany more intensively already during pharmacy studies (pharmacist) or vocational training (pharmaceutical technical assistant) using examples [30]. Another intervention option would be to expand the use of checklists, which so far exist for German CPs only for oral EC [81]. Supporting this, privacy should be ensured during counseling (e.g., through a separate counseling room), as this improves the exchange of information between customer and pharmacy staff [82]. In addition, regular, independent reviews of the quality of counseling should be conducted with an adequate sanction mechanism as an incentive [24].

### 4.2. Counseling for Self-Purchase and Purchase for a Third Party

In the special consideration of the quality of counseling, no significant differences between self-purchase and purchase for a third party with regard to the overall score were found in the present SPM study. With regard to the assumptions made in the literature, it has been shown that these could not be confirmed. Due to the lack of studies with the same object of investigation—as is not uncommon internationally [83] and limitedly acknowledged [84]—the present results cannot be compared.

However, it is clear that the question of the pharmacy staff “for whom” is very important to find out whether it is a self-purchase or a purchase for a third party. This question is foreseen in the BAK tool for self-medication for headache [18], but is not yet consistently asked in everyday pharmacy practice, as three German SPM studies for acute diarrhea [22, 55, 67] and two German nonparticipant observation studies [69, 85] could show. Pharmacy staff should therefore be encouraged to ask this question through appropriate measures—for example, training [30] and checklists [81]. In addition, customers should be sensitized through public campaigns to disclose right at the beginning of the consultation for whom the demand is. Moreover, in the case of purchase for a third party, third-party customers should be sensitized through public campaigns to gather information on the health status of the patient. This is important because relatively many third-party customers do not expect to be asked any questions because they have insufficient information about the patient and are reluctant to talk about medical problems of third parties [39], which would suggest that the quality of counseling must be poorer in the case of purchase for a third party. This is also supported by the fact that purchases for a third party occur much less frequently in everyday pharmacy practice [40, 41] and that pharmacy staff therefore have a lower routine in this respect. With regard to the provision of information, the pharmacy staff may be less motivated because they assume that not all the information given to the customer will be passed on to the third party. Moreover, the pharmacy staff may also have privacy concerns regarding the release of information. These reasons could explain the significantly poorer level of advice for an information item (“information about duration”) in the case of purchase for a third party.

On the other hand, it could be that the pharmacy staff provide better counseling to “compensate” for the assumed poor information transfer from the third-party customer to the patient. However, since in this study no significant differences between self-purchase and purchase for a third party were found overall, it is possible that the relevant motives for pharmacy staff balance each other or that no motives play a role at all. It would therefore be advisable to survey the pharmacy staff to clarify the motives. Subsequently, a customer survey would be useful to find out whether the motives named by the pharmacy staff actually apply from the customer's point of view. In addition, future studies should also examine whether the results of this study are transferable, for example, for other indications that are more “urgent” (e.g., EC), with the use of nonacademic SPs, with the specification of a younger or older third party or with an “extreme scenario” (e.g., necessary referral to a doctor).

### 4.3. Strengths and Limitations

As far as the authors are aware, this was the first study worldwide to analyze and report whether and to what extent differences exist between self-purchase and purchase for a third party in counseling by CPs. For this purpose, the SPM, referred to as the “gold standard” [86, 87], was used. However, it must be taken into account that the study was conducted in only one big city and for one indication. In addition, the results refer only to a specific point in time, since this is a cross-sectional study.

During implementation, each scenario was simulated 2 times in each CP (4 visits per CP), which may have increased the accuracy of the results. For the determination of possible differences in the quality of counseling it is essential for whom the purchase is. There could be a different counseling if, for example, a person at a young age asks for a person at an old age. In order to exclude bias in this respect as far as possible, the purchase for a third party was made for a person with a fairly similar age to the age of the respective SP.

When conducting the 4 visits in the same CP, it should be noted that the same pharmacy staff was not always encountered. However, this should not matter, as the CP owner should ensure a consistent level of counseling [88]. The 4 visits were conducted by different SPs, which on the one hand, in addition to minimizing the risk of detection, may have led to an averaging of the personal characteristics of the SPs and thus to the depiction of even more realistic counseling situations. On the other hand, it cannot be excluded that the different SPs had an influence on the results of the respective scenarios, which might have distorted the results. However, only objective items and dichotomous scales were used, so that the risk of intra- and interobserver variabilities typical for SPM studies was probably minimized.

With regard to quality assurance measures, (covert) audio or videotaping [89] was not used during the visits, as otherwise appropriate consent would have had to be obtained from the CPs in advance, which would have made it possible for the CPs not to participate in the study (opt-out), which in turn would probably have led to a selection bias [63]. A second observer [90] also had to be omitted due to the lack of human resources. Thus, recall bias due to faulty memories of the SPs cannot be excluded, but it could be minimized because the SPs filled out the assessment form directly after the respective visit outside the CP.

No specific measures were taken to find out whether visits were detected. This would have required the willingness of the investigated CPs to cooperate [91], which is likely to have been very limited due to the chosen study design (no opt-out). Data on the CP quality certificate and the professional group of the pharmacy staff were obtained in the context of self-reporting by the pharmacy staff through calls after completing all the visits, which means that deviations from the real situation cannot be excluded.

Asking CPs about reasons for inadequate counseling directly after each visit could have provided important additional information, but could also have jeopardized the covert study design (4 visits per CP). Another option is to collect this information after all visits have been completed, although there is then a risk of recall bias. Despite the fact that numerous control variables were collected, it cannot be excluded that other control variables—not used here—would have had an influence on the results of this study.

## 5. Conclusions

In view of the considerable deficits in the provision of advice by German CPs, which were also identified in this study, politicians and the regional chambers of pharmacists are called upon to take appropriate measures to improve the quality of counseling. Otherwise, CPs could run the risk that OTC medications in Germany—analogous to other countries—will be removed from the pharmacy-only obligation. It is positive that no differences in counseling were found between self-purchase and purchase for a third party, but further verifying studies with a modified methodology are recommended in this regard. In principle, pharmacy staff should be sensitized to ensure “adequate” counseling, regardless of the prevailing dialogue conditions.

## Figures and Tables

**Table 1:** Self-purchase scenario [27].

The SP entered the CP and said at the beginning of the conversation, “Hi, I need something for me for headache.” The SP did not have a particular product in mind. When questioned by the pharmacy staff, the following information was provided by the SP:	
Questions asked by the pharmacy staff What symptoms occur? How long have the symptoms been present? How often do the symptoms occur? Have other symptoms occurred? Are there other medical conditions? Which medications are taken regularly?	Information given by the SP Mild press headache on both sides Since yesterday From time to time No other symptoms No other medical conditions No other medications

**Table 2:** Purchase for a third-party scenario [27].

The SP entered the CP and said at the beginning of the conversation, “Hi, my boyfriend/girlfriend needs something for headache.” The SP did not have a particular product in mind. When questioned by the pharmacy staff, the following information was provided by the SP:	
Questions asked by the pharmacy staff What symptoms occur? How long have the symptoms been present? How often do the symptoms occur? Have other symptoms occurred? Are there other medical conditions? Which medications are taken regularly?	Information given by the SP Mild press headache on both sides Since yesterday From time to time No other symptoms No other medical conditions No other medications

**Table 3:** Assessment form.

Items		
	Yes	No
Possible questions asked by pharmacy staff:		
(1) What symptoms occur?	1	0
(2) How long have the symptoms been present?	1	0
(3) How often do the symptoms occur?	1	0
(4) Have other symptoms occurred?	1	0
(5) Are there other medical conditions?	1	0
(6) Which medications are taken regularly?	1	0
Possible information given by pharmacy staff:		
(7) Information about dosage	1	0
(8) Information about duration	1	0
(9) Information about side effects	1	0

**Table 4:** Control variables as well as time and type of data collection.

Control variables [literature source*∗*]	Time of data collection	Type of data collection
CP quality certificate [37]	After the visit	Exact measurement using a telephone query after completing all the visits
Gender of the SP [26]	Before the visit	Exact measurement based on the gender of the SP
Age of the SP [27]	Before the visit	Exact measurement based on the age of the SP
Gender of the pharmacy staff [33]	During the visit	Exact measurement using visual impression of the SP
Age of the pharmacy staff [32]	During the visit	Estimate using visual impression of the SP
Professional group of the pharmacy staff [34]	After the visit	Exact measurement based on the name tag and the receipt and, if necessary, using a telephone query by the SP after completing the visit
Time of the visit [29]	During the visit	Exact measurement using the SP's watch
Queue—customers waiting behind the SP [30]	During the visit	Exact measurement using visual impression of the SP
Active ingredient [29]	After the visit	Exact measurement using information of the package inserts

Note: *∗*The control variables were taken from the specific literature sources.

**Table 5:** CPs, SPs, pharmacy staff, visits, and active ingredients characteristics by scenario type.

	Total	Self-purchase scenario	Purchase for a third-party scenario	McNemar test *p* value (Cohen's *g*)
	*n* (%) 168 (100)	*n* (%) 84 (50.0)	*n* (%) 84 (50.0)	
CP quality certificate				
(i) No	76 (100)	38 (50.0)	38 (50.0)	n/a^#^
(ii) Yes	76 (100)	38 (50.0)	38 (50.0)	
(iii) Not able to be determined	16 (100)	8 (50.0)	8 (50.0)	

Gender of the SP				
(i) Male	84 (100)	24 (28.6)	60 (71.4)	<0.001*∗* (0.375)
(ii) Female	84 (100)	60 (71.4)	24 (28.6)	

Age of the SP				
(i) <30	102 (100)	72 (70.6)	30 (29.4)	<0.001*∗* (0.389)
(ii) 30–49	66 (100)	12 (18.2)	54 (81.8)	
(iii) ≥50	0 (0)	0 (0)	0 (0)	

Gender of the pharmacy staff				
(i) Male	30 (100)	11 (36.7)	19 (63.3)	0.077 (0.250)
(ii) Female	138 (100)	73 (52.9)	65 (47.1)	

Age of the pharmacy staff				
(i) <30	16 (100)	9 (56.3)	7 (43.7)	0.947^#^
(ii) 30–49	91 (100)	45 (49.5)	46 (50.5)	
(iii) 50	61 (100)	30 (49.2)	31 (50.8)	

Professional group of the pharmacy staff				
(i) Pharmacist	89 (100)	44 (49.4)	45 (50.6)	0.773^#^
(ii) Nonpharmacist	72 (100)	37 (51.4)	35 (48.6)	
(iii) Not able to be determined	7 (100)	3 (42.9)	4 (57.1)	

Time of the visit				
(i) 8:00 a.m.–12:00 p.m.	26 (100)	13 (50.0)	13 (50.0)	0.003^#^*∗*
(ii) 12:01 p.m.–4:00 p.m.	85 (100)	54 (63.5)	31 (36.5)	
(iii) 4:01 p.m.–8:00 p.m.	57 (100)	17 (29.8)	40 (70.2)	

Queue—customers waiting behind the SP				
(i) No	120 (100)	67 (55.8)	53 (44.2)	0.029*∗* (0.194)
(ii) Yes	48 (100)	17 (35.4)	31 (64.6)	

Active ingredient				
(iii) Paracetamol	34 (100)	23 (67.6)	11 (32.4)	0.153^#^
(iv) Ibuprofen	101 (100)	46 (45.5)	55 (54.5)	
(v) Others	33 (100)	15 (45.5)	18 (54.5)	

^#^ McNemar–Bowker test; n/a (not applicable); *∗*significant at *p* < 0.05.

**Table 6:** Assessment items and counseling score by scenario type.

		Total	Self-purchase scenario	Purchase for a third-party scenario	McNemar test *p* value (Cohen's *g*)
		*n* (%) 168 (100)	*n* (%) 84 (50.0)	*n* (%) 84 (50.0)	
(1) What symptoms occur?					
(i) No		107 (100)	51 (47.7)	56 (52.3)	0.487 (0.076)
(ii) Yes		61 (100)	33 (54.1)	28 (45.9)	

(2) How long have the symptoms been present?					
(i) No		151 (100)	74 (49.0)	77 (51.0)	0.607 (0.100)
(ii) Yes		17 (100)	10 (58.8)	7 (41.2)	

(3) How often do the symptoms occur?					
(i) No		139 (100)	67 (48.2)	72 (51.8)	0.424 (0.077)
(ii) Yes		29 (100)	17 (58.6)	12 (41.4)	

(4) Have other symptoms occurred?					
(i) No		124 (100)	66 (53.2)	58 (46.8)	0.200 (0.133)
(ii) Yes		44 (100)	18 (40.9)	26 (59.1)	

(5) Are there other medical conditions?					
(i) No		79 (100)	46 (58.2)	33 (41.8)	0.066 (0.151)
(ii) Yes		89 (100)	38 (42.7)	51 (57.3)	

(6) Which medications are taken regularly?					
(i) No		100 (100)	49 (49.0)	51 (51.0)	0.868 (0.028)
(ii) Yes		68 (100)	35 (51.5)	33 (48.5)	

(7) Information about dosage given					
(i) No		44 (100)	23 (52.3)	21 (47.3)	0.864 (0.029)
(ii) Yes		124 (100)	61 (49.2)	63 (50.8)	

(8) Information about duration given					
(i) No		105 (100)	43 (41.0)	62 (59.0)	0.003^*∗*^ (0.244)
(ii) Yes		63 (100)	41 (65.1)	22 (34.9)	

(9) Information about side effects given					
(i) No		159 (100)	78 (49.1)	81 (50.9)	0.453 (0.214)
(ii) Yes		9 (100)	6 (66.7)	3 (33.3)	

Counseling score	Mean (SD)	3.0 (1.8)	3.1 (1.9)	2.9 (1.7)	0.495^#^ (0.053)
	Median (IQR)	3.0 (2.0)	3.0 (2.0)	3.0 (2.0)	

^#^ Wilcoxon signed-rank test (Pearson's *r*); ^*∗*^significant at *p* < 0.05.

**Table 7:** Association between counseling level and scenario type.

Variables	Total	Counseling level below average	Counseling level above average	COR (95% CI)	*p* value	AOR (95% CI)	*p* value
	*n* (%) 168 (100)	*n* (%) 134 (79.8)	*n* (%) 34 (20.2)				
Scenario type							
(i) Self-purchase	84 (100)	66 (78.6)	18 (21.4)	1	0.701	1	0.278
(ii) Purchase for a third party	84 (100)	68 (81.0)	16 (19.0)	0.863 (0.406–1.833)		1.635 (0.673–3.972)	

Control variables							
CP quality certificate							
(i) No	76 (100)	55 (72.4)	21 (27.6)	1		1	
(ii) Yes	76 (100)	65 (85.5)	11 (14.5)	0.443 (0.197–0.999)	0.049^*∗*^	0.435 (0.188–1.006)	0.052
(iii) Not able to be determined	16 (100)	14 (87.5)	2 (12.5)	0.374 (0.078–1.789)	0.218	0.329 (0.067–1.615)	0.171

Gender of the SP							
(i) Male	84 (100)	73 (86.9)	11 (13.1)	1		1	0.779
(ii) Female	84 (100)	61 (72.6)	23 (27.4)	2.502 (1.130–5.540)	0.024^*∗*^	1.194 (0.346–4.126)	

Age of the SP							
(i) <30	102 (100)	75 (73.5)	27 (26.5)	1		1	0.090
(ii) 30–49	66 (100)	59 (89.4)	7 (10.6)	0.330 (0.134–0.809)	0.015^*∗*^	0.283 (0.066–1.217)	
(iii) ≥50	0 (0)	0 (0)	0 (0)	—	—	—	—

Gender of the pharmacy staff							
(i) Male	30 (100)	27 (90.0)	3 (10.0)	1	0.135		
(ii) Female	138 (100)	107 (72.5)	31 (22.5)	2.607 (0.741–9.174)			

Age of the pharmacy staff							
(i) <30	16 (100)	11 (68.7)	5 (31.3)	1			
(ii) 30–49	91 (100)	70 (76.9)	21 (23.1)	0.660 (0.206–2.114)	0.484		
(iii) ≥50	61 (100)	53 (86.9)	8 (13.1)	0.332 (0.091–1.209)	0.085		

Professional group of the pharmacy staff							
(i) Pharmacist	89 (100)	71 (79.8)	18 (20.2)	1			
(ii) Nonpharmacist	72 (100)	56 (77.8)	16 (22.2)	1.127 (0.528–2.408)	0.758		
(iii) Not able to be determined	7 (100)	7 (100)	0 (0)	0 (0)	0.999		

Time of the visit							
(i) 8:00 a.m.–12:00 p.m.	26 (100)	21 (80.8)	5 (19.2)	1			
(ii) 12:01 p.m.–4:00 p.m.	85 (100)	66 (77.6)	19 (22.4)	1.209 (0.402–3.635)	0.735		
(iii) 4:01 p.m.–8:00 p.m.	57 (100)	47 (82.5)	10 (17.5)	0.894 (0.272–2.939)	0.853		

Queue—customers waiting behind the SP							
(i) No	120 (100)	92 (76.7)	28 (23.3)	1			
(ii) Yes	48 (100)	42 (87.5)	6 (12.5)	0.469 (0.181–1.219)	0.120		

Active ingredient							
(i) Paracetamol	34 (100)	27 (79.4)	7 (20.6)	1			
(ii) Ibuprofen	101 (100)	76 (75.2)	25 (24.8)	1.269 (0.493–3.268)	0.622		
(iii) Others	33 (100)	31 (93.9)	2 (6.1)	0.249 (0.048–1.301)	0.099		

COR: crude odds ratio; AOR adjusted odds ratio. ^*∗*^significant at *p* < 0.05.

## Data Availability

The datasets are available from the corresponding author upon reasonable request.

## References

[1] GBD 2017 Disease and Injury Incidence and Prevalence Collaborators (2018). Global, regional, and national incidence, prevalence, and years lived with disability for 354 diseases and injuries for 195 countries and territories, 1990–2017: a systematic analysis for the Global Burden of Disease Study 2017. *Lancet*.

[2] GBD 2016 Headache Collaborators (2018). Global, regional, and national burden of migraine and tension-type headache, 1990–2016: a systematic analysis for the Global Burden of Disease Study 2016. *The Lancet Neurology*.

[3] Linde M., Gustavsson A., Stovner L. J. (2012). The cost of headache disorders in Europe: the Eurolight project. *European Journal of Neurology*.

[4] Seddik A. H., Branner J. C., Ostwald D. A., Schramm S. H., Bierbaum M., Katsarava Z. (2020). The socioeconomic burden of migraine: an evaluation of productivity losses due to migraine headaches based on a population study in Germany. *Cephalalgia*.

[5] Paudyal V., Cunningham S., Smith K. G., MacLure K., Ryan C., Cordina M. (2018). Methodological considerations in clinical outcomes assessment of pharmacy-based minor ailments management: a systematic review. *PLoS One*.

[6] Yusuff K. B., Makhlouf A. M., Ibrahim M. I. (2021). Community pharmacists’ management of minor ailments in developing countries: a systematic review of types, recommendations, information gathering and counseling practices. *International Journal of Clinical Practice*.

[7] Shehnaz S. I., Agarwal A. K., Khan N. (2014). A systematic review of self-medication practices among adolescents. *Journal of Adolescent Health*.

[8] Limaye D., Limaye V., Krause G. (2017). A systematic review of the literature to assess self-medication practices. *Annals of Medical and Health Sciences Research*.

[9] Eichenberg C., Auersperg F., Rusch B. D., Brähler E. (2015). Self-medication: a nationwide representative survey on motives, reasons and sources on consuming over-the-counter medication. *Psychotherapie, Psychosomatik, Medizinische Psychologie*.

[10] Haag G., Diener H.-C., May A. (2011). Self-medication of migraine and tension-type headache: summary of the evidence-based recommendations of the Deutsche Migräne und Kopfschmerzgesellschaft (DMKG), the Deutsche Gesellschaft für Neurologie (DGN), the Österreichische Kopfschmerzgesellschaft (ÖKSG) and the Schweizerische Kopfwehgesellschaft (SKG). *The Journal of Headache and Pain*.

[11] Straube A. (2015). *Therapy of episodic and chronic tension-type headache and other chronic daily headache*.

[12] Moore R. A., Wiffen P. J., Derry S., Maguire T., Roy Y. M., Tyrrell L. (2015). Non-prescription (OTC) oral analgesics for acute pain–an overview of Cochrane reviews. *Cochrane Database of Systematic Reviews*.

[13] Perrot S., Cittée J., Louis P. (2019). Self-medication in pain management: the state of the art of pharmacists’ role for optimal Over-The-Counter analgesic use. *European Journal of Pain*.

[14] Oleszkiewicz P., Krysinski J., Religioni U., Merks P. (2021). Access to medicines via non-pharmacy outlets in European countries-A review of regulations and the influence on the self-medication phenomenon. *Healthcare*.

[15] Grunenberg M., Bäumler M. (2021). Zurück in die Zukunft: Zur Modernisierung der Arzneimittelversorgung durch Apotheken [Back to the Future: On the Modernisation of Pharmaceutical Supply by Pharmacies]. *Gesundheits- und Sozialpolitik*.

[16] Baratta F., Allais G., Rolando S. (2019). Prevention, education and counselling: the worldwide role of the community pharmacist as an epidemiological sentinel of headaches. *Neurological Sciences*.

[17] ApBetrO (2016). *Ordinance on the Operation of Pharmacies in the Version as Published on September 26, 1995 (Federal Law Gazette I P. 1195), Last Amended Pursuant to Article 1a of the Law for the Implementation of Directives (EU) 2015/566 and (EU) 2015/565 on the Import and the Coding of Human Tissues and Tissues Preparations on November 21, 2016 (Federal Law Gazette I p. 2623)*.

[18] BAK–Bundesapothekerkammer (2019). *Information und Beratung im Rahmen der Selbstmedikation am Beispiel Kopfschmerzen [Information and Advice in the Context of Self-Medication Using the Example of Headaches]*.

[19] Barrenberg E., Garbe E. (2015). Use of over-the-counter (OTC) drugs and perceptions of OTC drug safety among German adults. *European Journal of Clinical Pharmacology*.

[20] Berger K., Eickhoff C., Schulz M. (2005). Counseling quality in community pharmacies: implementation of the pseudo customer methodology in Germany. *Journal of Clinical Pharmacy and Therapeutics*.

[21] Alte D., Weitschies W., Ritter C. A. (2007). Evaluation of consultation in community pharmacies with mystery shoppers. *The Annals of Pharmacotherapy*.

[22] Langer B., Kunow C. (2020). Do north-eastern German pharmacies recommend a necessary medical consultation for acute diarrhoea? Magnitude and determinants using a simulated patient approach. *F1000Research*.

[23] Langer B., Grimm S., Lungfiel G., Mandlmeier F., Wenig V. (2020). The quality of counselling for oral emergency contraceptive pills—A simulated patient study in German community pharmacies. *International Journal of Environmental Research and Public Health*.

[24] Kunow C., Langer B. (2021). Using the simulated patient methodology to assess the quality of counselling in German community pharmacies: a systematic review from 2005 to 2018. *International Journal of Pharmacy and Pharmaceutical Sciences*.

[25] Zapata-Cachafeiro M., Piñeiro-Lamas M., Guinovart M. C., López-Vázquez P., Vázquez-Lago J. M., Figueiras A. (2019). Magnitude and determinants of antibiotic dispensing without prescription in Spain: a simulated patient study. *Journal of Antimicrobial Chemotherapy*.

[26] Paravattil B., Kheir N., Yousif A. (2017). Utilization of simulated patients to assess diabetes and asthma counseling practices among community pharmacists in Qatar. *International Journal of Clinical Pharmacy*.

[27] Kunow C., Langer B. (2021). Dispensing and variabilities in pricing of headache OTC medicines by community pharmacies in a German big city: a simulated patient Approach. *ClinicoEconomics and Outcomes Research*.

[28] Horvat N., Koder M., Kos M. (2012). Using the simulated patient methodology to assess paracetamol-related counseling for headache. *PLoS One*.

[29] Kashour T. S., Joury A., Alotaibi A. M. (2016). Quality of assessment and counseling offered by community pharmacists and medication sale without prescription to patients presenting with acute cardiac symptoms: a simulated client study. *European Journal of Clinical Pharmacology*.

[30] Langer B., Bull E., Burgsthaler T., Glawe J., Schwobeda M., Simon K. (2016). Using the simulated patient methodology to assess counseling for acute diarrhoea–evidence from Germany. *Zeitschrift für Evidenz, Fortbildung und Qualität im Gesundheitswesen*.

[31] Uzun G. D., Sancar M., Okuyan B. (2019). Evaluation of knowledge and attitude of pharmacist and pharmacy technicians on emergency contraception method in Istanbul, Turkey: a simulated patient study. *Journal of Research in Pharmacy*.

[32] Mináriková D., Fazekaš T., Minárik P., Jurišová E. (2019). Assessment of patient counseling on the common cold treatment at Slovak community pharmacies using mystery shopping. *Saudi Pharmaceutical Journal*.

[33] Saba M., Diep J., Bittoun R., Saini B. (2014). Provision of smoking cessation services in Australian community pharmacies: a simulated patient study. *International Journal of Clinical Pharmacy*.

[34] Collins J. C., Schneider C. R., Naughtin C. L., Wilson F., de Almeida Neto A. C., Moles R. J. (2017). Mystery shopping and coaching as a form of audit and feedback to improve community pharmacy management of non-prescription medicine requests: an intervention study. *BMJ Open*.

[35] Byrne G. A., Wood P. J., Spark M. J. (2018). Non-prescription supply of combination analgesics containing codeine in community pharmacy: a simulated patient study. *Research in Social and Administrative Pharmacy*.

[36] Llor C., Monnet D. I., Cots J. M. (2010). Small pharmacies are more likely to dispense antibiotics without a medical prescription than large pharmacies in Catalonia, Spain. *Euro Surveillance*.

[37] Kippist C., Wong K., Bartlett D., Saini B. (2011). How do pharmacists respond to complaints of acute insomnia? a simulated patient study. *International Journal of Clinical Pharmacy*.

[38] Jackson R. A., Smith M. C. (1974). Relations between price and quality in community pharmacy. *Medical Care*.

[39] Morris C. J., Cantrill J. A., Weiss M. C. (1997). One simple question should be enough”: consumers’ perceptions of pharmacy protocols. *International Journal of Pharmacy Practice*.

[40] Collins J. C., Schneider C. R., El-Den S., Moles R. J. (2020). Self-care-seeking behaviors in the community pharmacy: a cross-sectional exit survey of Australian consumers. *Journal of the American Pharmacists Association*.

[41] Anyama N., Adome R. O. (2003). Community pharmaceutical care: an 8-month critical review of two pharmacies in Kampala. *African Health Sciences*.

[42] BAK-Bundesapothekerkammer (2019). *Kommentar zur Leitlinie der Bundesapothekerkammer zur Qualitätssicherung: Information und Beratung des Patienten bei der Abgabe von Arzneimitteln – Selbstmedikation [Commentary on the Guideline of the German Federal Chamber of Pharmacists on Quality Assurance: Information and Advice to the Patient when Dispensing Medicinal Products – Self-medication]*.

[43] PSA–Pharmaceutical Society of Australia (2017). *Professional Practice Standards Version 5*.

[44] Dierolf V., Freytag S. (2017). Zugang zur Pille danach in den Apotheken nach der Rezeptfreigabe. [Access to the morning-after pill in pharmacies after release from prescription-only status]. *Pro Familia Magazin*.

[45] Chowdhury F., Sturm-Ramirez K., Al Mamun A. (2018). Effectiveness of an educational intervention to improve antibiotic dispensing practices for acute respiratory illness among drug sellers in pharmacies, a pilot study in Bangladesh. *BMC Health Services Research*.

[46] Xu T., de Almeida Neto A. C., Moles R. J. (2012). A systematic review of simulated-patient methods used in community pharmacy to assess the provision of non-prescription medicines. *International Journal of Pharmacy Practice*.

[47] Björnsdottir I., Granas A. G., Bradley A., Norris P. (2020). A systematic review of the use of simulated patient methodology in pharmacy practice research from 2006 to 2016. *International Journal of Pharmacy Practice*.

[48] da Costa F. A., Babar Z. U. D. (2020). Covert and overt observations in pharmacy practice. *Pharmacy Practice Research Methods*.

[49] STROBE Statement (2021). *Checklist of Items that Should Be Included in Reports of Cross-Sectional Studies*.

[50] Amaratunge S., Harrison M., Clifford R., Seubert L., Page A., Bond C. (2021). Developing a checklist for reporting research using simulated patient methodology (CRiSP): a consensus study. *International Journal of Pharmacy Practice*.

[51] Statistisches Bundesamt (2021). *Daten aus dem Gemeindeverzeichnis. Städte in Deutschland nach Fläche, Bevölkerung und Bevölkerungsdichte [Data from the Municipal Directory. Cities in Germany by Area, Population and Population Density]*.

[52] Landesapothekerkammer Brandenburg (2020). *Apothekendatenbank im Land Brandenburg [Pharmacy database in the federal state of Brandenburg]*.

[53] Apotheken-Umschau *Apothekensuche: Finden Sie Apotheken in Ihrer Nähe! [Pharmacy Search: Find Pharmacies Near You!]*.

[54] IHS–Headache Classification Committee of the International Headache Society (2018). The international classification of headache disorders. *Cephalalgia*.

[55] Langer B., Bull E., Burgsthaler T., Glawe J., Schwobeda M., Simon K. (2018). Assessment of counseling for acute diarrhoea in German pharmacies: a simulated patient study. *International Journal of Pharmacy Practice*.

[56] Ahmed T., Assefa N., Demisie A., Kenay A. (2014). Levels of adult patients’ satisfaction with nursing care in selected public hospitals in Ethiopia. *International Journal of Health Sciences*.

[57] Kasa A. S., Gedamu H. (2019). Predictors of adult patient satisfaction with nursing care in public hospitals of Amhara region, Northwest Ethiopia. *BMC Health Services Research*.

[58] Cohen J. (1992). A power primer. *Psychological Bulletin*.

[59] Tesfaye B., Tewabe M., Ferede A., Dawson A. (2020). Induced second trimester abortion and associated factors at debre Markos referral hospital: cross-sectional study. *Women’s Health*.

[60] Zegeye B., Keetile M., Ahinkorah B. O., Kwabena Ameyaw E., Seidu A.-A., Yaya S. (2021). Association between attitude towards wife beating and childhood diarrhea: a demographic and health survey-based study in 25 sub-saharan african countries. *The Scientific World Journal*.

[61] ADM–Arbeitskreis Deutscher Markt- und Sozialforschungsinstitute e.V. (2006). *Richtlinie für den Einsatz von Mystery Research in der Markt- und Sozialforschung [Guideline for the Use of Mystery Research in Market and Social Research]*.

[62] McCambridge J., Witton J., Elbourne D. R. (2014). Systematic review of the Hawthorne effect: new concepts are needed to study research participation effects. *Journal of Clinical Epidemiology*.

[63] Rhodes K. V., Miller F. G. (2012). Simulated patient studies: an ethical analysis. *The Milbank Quarterly*.

[64] Kayshap K. C., Nissen L. M., Smith S. S., Kyle G. (2014). Management of over-the-counter insomnia complaints in Australian community pharmacies: a standardized patient study. *International Journal of Pharmacy Practice*.

[65] da Rocha C. E., Bispo M. L., dos Santos A. C., Mesquita A. R., Brito G. C., de Lyra D. P. (2015). Assessment of community pharmacists’ counseling practices with simulated patients who have minor illness: a pilot study. *Simulation in Healthcare*.

[66] Mobark D. M., Al-Tabakha M. M., Hasan S. (2019). Assessing hormonal contraceptive dispensing and counseling provided by community pharmacists in the United Arab Emirates: a simulated patient study. *Pharmacy Practice*.

[67] Langer B., Kieper M., Laube S., Schramm J., Weber S., Werwath A. (2018). Assessment of counselling for acute diarrhoea in North-Eastern German pharmacies – a follow-up study using the simulated patient methodology. *Pharmacology & Pharmacy*.

[68] Schumacher P. M., Neininger M. P., Kaune A., Bertsche T. (2019). Counseling patients on correct drug handling in German community pharmacies: experiences and opinions of pharmaceutical staff. *International Journal of Clinical Pharmacy*.

[69] Seiberth J. M., Moritz K., Kücükay N., Schiek S., Bertsche T. (2020). What is the attitude towards and the current practice of information exchange during self-medication counselling in German community pharmacies? an assessment through self-report and non-participant observation. *PLoS One*.

[70] Seiberth J. M., Moritz K., Vogel C. F., Bertsche T., Schiek S. (2021). Public’s perspectives on guideline-recommended self-medication consultations in German community pharmacies. *Health and Social Care in the Community*.

[71] MacFarlane B. V., Bergin J. K., Reeves P., Matthews A. (2015). Australian pharmacies prevent potential adverse reactions in patients taking warfarin requesting over-the-counter analgesia. *International Journal of Pharmacy Practice*.

[72] Mesquita A. R., Bezerra de Oliveira Sá D. A., Santos A. P., de Almeida Neto A., Lyra D. P. (2013). Assessment of pharmacist’s recommendation of non-prescription medicines in Brazil: a simulated patient study. *International Journal of Clinical Pharmacy*.

[73] Santos A. P., Mesquita A. R., Oliveira K. S., Lyra D. P. (2013). Assessment of community pharmacists’ counselling skills on headache management by using the simulated patient approach: a pilot study. *Pharmacy Practice*.

[74] Hammad E. A., Elayeh E., Tubeileh R., Watson M., Wazaify M. (2018). A simulated patient study assessing over the counter supply and counseling in Jordan: responding to headache complaints. *International Journal of Clinical Pharmacy*.

[75] Netere A. K., Erku D. A., Sendekie A. K., Gebreyohannes E. A., Muluneh N. Y., Belachew S. A. (2018). Assessment of community pharmacy professionals’ knowledge and counseling skills achievement towards headache management: a cross-sectional and simulated-client based mixed study. *The Journal of Headache and Pain*.

[76] Kelly F. S., Williams K. A., Benrimoj S. I. (2009). Does advice from pharmacy staff vary according to the nonprescription medicine requested?. *The Annals of Pharmacotherapy*.

[77] Gastelurrutia M. A., Larrañaga B., Garay A., de Asís Echeveste F., Fernandez-Llimos F. (2013). Impact of a program to reduce the dispensing of antibiotics without a prescription in Spain. *Pharmacy Practice*.

[78] Pham D. M., Byrkit M., Van Pham H., Pham T., Nguyen C. T. (2013). Improving pharmacy staff knowledge and practice on childhood diarrhea management in Vietnam: are educational interventions effective?. *PLoS One*.

[79] Foroughinia F., Zarei P. (2016). Evaluation of knowledge, attitude, and practice of community pharmacists toward administration of over-the-counter drugs for the treatment of diarrhea in children: a pretest-posttest survey. *Journal of Research in Pharmacy Practice*.

[80] Marković-Peković V., Grubiša N., Burger J., Bojanić L., Godman B. (2017). Initiatives to reduce nonprescription sales and dispensing of antibiotics: findings and implications. *Journal of Research in Pharmacy Practice*.

[81] BAK–Bundesapothekerkammer (2021). *Rezeptfreie Abgabe von Notfallkontrazeptiva (“Pille danach”). Handlungsempfehlungen der Bundesapothekerkammer. [Dispensing of emergency contraceptives (“morning-after pill”) without prescription. Recommendations for action by the German Federal Chamber of Pharmacists]*.

[82] Seubert L. J., Whitelaw K., Boeni F., Hattingh L., Watson M. C., Clifford R. M. (2017). Barriers and facilitators for information exchange during over-the-counter consultations in community pharmacy: a focus group study. *Pharmacy*.

[83] Yaffe J., Montgomery P., Hopewell S., Shepard L. D. (2012). Empty reviews: a description and consideration of Cochrane systematic reviews with no included studies. *PLoS One*.

[84] Awoke M., Melaku T., Beshir M. (2021). Drug-related problems and its determinant among hospitalized neonates with sepsis at Jimma University Medical Center, Ethiopia: a prospective observational study. *Journal of Pharmaceutical Health Care and Sciences*.

[85] Seiberth J. M., Moritz K., Herrmann N. S., Bertsche T., Schiek S. (2022). What influences the information exchange during self-medication consultations in community pharmacies? a non-participant observation study. *Research in Social and Administrative Pharmacy*.

[86] Shah R., Edgar D., Evans B. J. W. (2007). Measuring clinical practice. *Ophthalmic and Physiological Optics*.

[87] Collins J. C., Chong W. W., de Almeida Neto A. C., Moles R. J., Schneider C. R. (2021). The simulated patient method: design and application in health services research. *Research in Social and Administrative Pharmacy*.

[88] Langer B., Kunow C. (2019). Medication dispensing, additional therapeutic recommendations, and pricing practices for acute diarrhoea by community pharmacies in Germany: a simulated patient study. *Pharmacy Practice*.

[89] Werner J. B., Benrimoj S. I. (2008). Audio taping simulated patient encounters in community pharmacy to enhance the reliability of assessments. *American Journal of Pharmaceutical Education*.

[90] Khojah H. M. J. (2019). Privacy level in private community pharmacies in Saudi arabia: a simulated client survey. *Pharmacology & Pharmacy*.

[91] Obreli-Neto P. R., Pereira L. R. L., Guidoni C. M. (2013). Use of simulated patients to evaluate combined oral contraceptive dispensing practices of community pharmacists. *PLoS One*.

